# Supplementary material for: The genome sequence of the White-speckled Fungus moth, Nemapogon koenigi Capuse, 1967 (Lepidoptera: Tineidae)
Source: Wellcome Open Res. 2025 Oct 13;10:569. [Version 1] doi: 10.12688/wellcomeopenres.24972.1 (PMC12728341; doi:10.12688/wellcomeopenres.24972.1)
Supplement: Peer review history

# Reviewer report: Reviewer response for version 1

**DOI:** 10.21956/wellcomeopenres.27507.r137983

**Published:** 2025-12-23

**Authors:** Marko Mutanen

**Affiliations:** 1 University of Oulu, Oulu, Finland

This article Index a chromosome-level genome for a moth Nemapogon koenigi of the family Tineidae. Tineids are primitive ditrysian Lepidoptera, and to understand the early evolution of this massive radiation, genomes more species of them are needed. Tineid moths are also unusual lepidopteran regarding their diet as they are seldom herbivores.

 The species introduction is well-prepared. I appreciate that it also gives a short account also on the phylogenetics and classification of the species in question (this is often absent in similar reports). In the first paragraph, the statement of: "...it is not generally regarded as a household textile pest..." appears a bit odd. Why would a species feeding on fungi in dead wood ever be regarded as a textile pest? That some other (rather distantly related) species of Tineidae feed on animal fibers (keratin) should not be used to blame the whole family. I think the word "Ditrysian" in the introduction should not be capitalized as being an adjective (like lepidopteran).

 The genome itself is, as far as I can see, of very high-quality. All the provided statistics and graphs, such as the high BUSCO recovery rate, demonstrate that the genome provides a reliable reference to N. koenigi. The identification was confirmed through barcoding which makes me confident that the specimen is correctly identified (species of this genus are frequently tricky to identify).

Are sufficient details of methods and materials provided to allow replication by others?

Yes

Is the rationale for creating the dataset(s) clearly described?

Yes

Are the datasets clearly presented in a useable and accessible format?

Yes

Are the protocols appropriate and is the work technically sound?

Yes

Reviewer Expertise:

Lepidoptera systematics, phylogenetics, genomics, DNA barcoding, taxonomy, dark taxa

I confirm that I have read this submission and believe that I have an appropriate level of expertise to confirm that it is of an acceptable scientific standard.

# Reviewer report: Reviewer response for version 1

**DOI:** 10.21956/wellcomeopenres.27507.r137982

**Published:** 2025-11-25

**Authors:** Panagiotis Ioannidis

**Affiliations:** 1 Foundation for Research & Technology - Hellas, Crete, Greece

This paper by Langdon et al describes the sequencing and assembly of the genome of the lepidopteran Nemapogon koenigi.

 The background information provided in the Introduction give a good enough description of the evolution of this species. That said, however, I've seen more detailed background information in other genome projects.

 The methodology is the standard one in all DToL projects and yielded a very contiguous assembly. More specifically, scaffold N50 is 13 Mbp, >99% of the assembly is found in one of the 31 chromosomal level scaffolds, CC ratio = 52/31 = 1.67, k-mer completeness is >99%, QV = 58.8, and complete BUSCO = 96%. In addition the blobplot (Figure 6) looks very "clean" with virtually no other scaffolds, other than the 31 chromosome-level ones (which is in agreement with the very good CC ratio). Finally it is nice to see that genome annotation is also scheduled to be done in Ensembl, thus making it easy for everyone to use.

 In summary the herein presented genome assembly is an excellent one. My only (minor) criticism would be that I'd like to see additional background information for the ecology and life cycle of this species.

Are sufficient details of methods and materials provided to allow replication by others?

Yes

Is the rationale for creating the dataset(s) clearly described?

Yes

Are the datasets clearly presented in a useable and accessible format?

Yes

Are the protocols appropriate and is the work technically sound?

Yes

Reviewer Expertise:

insect genomics; bioinformatics

I confirm that I have read this submission and believe that I have an appropriate level of expertise to confirm that it is of an acceptable scientific standard.
